# Dry Needling in Overhead Athletes with Myofascial Shoulder Pain: A Systematic Review

**DOI:** 10.3390/sports12060156

**Published:** 2024-06-05

**Authors:** Andrea Demeco, Alessandro de Sire, Antonello Salerno, Nicola Marotta, Stefano Palermi, Antonio Frizziero, Cosimo Costantino

**Affiliations:** 1 Department of Medicine and Surgery, University of Parma, 43126 Parma, Italy; andrea.demeco@unipr.it (A.D.); antonio.frizziero@unipr.it (A.F.); cosimo.costantino@unipr.it (C.C.); 2 Physical and Rehabilitative Medicine, Department of Experimental and Clinical Medicine, University of Catanzaro “Magna Graecia”, 88100 Catanzaro, Italy; alessandro.desire@unicz.it (A.d.S.); nicola.marotta@unicz.it (N.M.); 3 Research Center on Musculoskeletal Health, MusculoSkeletalHealth@UMG, University of Catanzaro “Magna Graecia”, 88100 Catanzaro, Italy; 4 Public Health Department, University of Naples Federico II, 80131 Naples, Italy; stefano.palermi@unina.it; 5 ASST “Gaetano Pini” CTO, 20122 Milano, Italy

**Keywords:** dry needling, volleyball, baseball, tennis, sports, rehabilitation, pain management

## Abstract

The treatment of myofascial pain in athletes requires a set of rehabilitation techniques that aim to be effective quickly. In this context, dry needling (DNY) has shown interesting results due to its ability to reduce pain in the short term. Thus, the aim of this study was to investigate the role of DNY in managing myofascial shoulder pain in overhead athletes. PubMed, Scopus and Web of Science were screened up to March 2024, to identify studies that met the following inclusion criteria: overhead athletes with shoulder pain with a DNY approach for myofascial trigger points (MTrPs), RCT, case–control study, feasibility study as the study design. Exclusion criteria were studies that did not include athletes, studies that did not focus on the treatment of MTrPs with DNY, other reviews, no full-text availability and papers written in a language other than English. Out of 399 articles, 165 were excluded as duplicates. Of the 234 articles screened, only 6 articles met the inclusion criteria. A total of 6 studies were included in the systematic review. Initial results showed that DNY improved pain rapidly and in the short term; however, there is still no consensus on the minimum number and the interval between treatments. Major findings reported a rapid potential decrease in perceived pain, shoulder disability and an increase in muscle strength; in this scenario, DNY might be a valid solution in a sports rehabilitation setting.

## 1. Introduction

Shoulder pain is a prevalent and debilitating musculoskeletal disorder among overhead athletes [1,2,3,4]. Explosive and repetitive movements over the head can lead to overuse injuries in shoulders, resulting in extended periods out of competition in sportsmen [5,6,7,8,9,10]. The prevalence and incidence of shoulder injury in overhead sports range from 5% to 36% and 0.2/1000 to 1.8/1000 h [3,11,12], respectively. However, as Mohseni-Bandpeiet et al. reported [13], the high heterogeneity of the prevalence depends on the different sport, athletes’ age, gender, sport level and training loads.

Lo et al. [14], analyzing shoulder pathologies among various sports, reported that almost half of athlete experience shoulder disorders, and one third demonstrated pain. These issues were more prevalent in volleyball and swimming, particularly in elite categories; inasmuch the most common shoulder pathologies include scapular dyskinesia, impingement syndrome, superior labrum anterior to posterior (SLAP) tears, glenohumeral internal rotation deficit (GIRD), rotator cuff tears and myofascial trigger points (MTrPs) [15,16,17,18,19,20]. MTrPs, in particular, account for musculoskeletal pain in 30% of patients with musculoskeletal disorders; on the other hand, in high-level athletes, myofascial syndrome pain might significantly reduce performance [21]. MTrPs are defined as irritable points within a tense band of skeletal muscle, which cause pain during stretching, contraction, or palpation. These points can lead to postural changes, reduced movement, and muscle weakness or stiffness [16,22].

Clinically, MTrPs are caused by an acute or chronic muscle overload as a result of repetitive movements or prolonged muscle contraction [23]. Simon et al. [18] suggested that the formation of the tense band is due to an alteration in the potential of the secondary terminal plaque, with an excessive release of acetylcholine at the neuromuscular junction. This theory supports a high “noise of the terminal plate” which explains the persistent presence of contractures at the sarcomere level, leading to local ischemia and hypoxia, and consequently, pain [24]. Moreover, the release of algogenic and vasoactive substances in the muscle stimulates peripheral nociceptors (peripheral sensitivity), and the neurons of the central horn and supra-spinal structures, resulting in hyperalgesia, allodynia, and related pain (central sensitization) [18,25,26].

MTrPs can be divided into Active (ATrPs) or Latent (LTrPs): ATrPs spontaneously produce pain regardless of activity, and can clinically be associated with a local twitch response (LTR) (a brief and sudden contraction of the TrP tight band upon palpation or needle insertion and thought to be a spinal reflex associated with hyperexcitability of dysfunctional motor endplates). On the other hand, LTrPs cause pain only if stimulated manually or by a needle [16,27].

In this scenario, the most affected muscles are the upper trapezius (UT), the infraspinatus (ISP) and teres major muscles. In particular, ATrPs in the UT can induce pain in the neck, shoulder, headache and dizziness in addition to local pain, while ATrPa in the ISP can produce shoulder-related pain by mimicking cervical radiculopathic pain [16,28] that could overlap the symptomatology related to other conditions such as bursitis, tendinopathies, fibromyalgia and fasciitis [27].

Treatment options might include manual therapy (e.g., massage, stretching, sleeper stretch, passive rhythmic release, active rhythmic release, trigger point pressure release, fascial manipulation), supervised throwing exercise, self-exercise program, plyometric exercises, muscle strengthening exercises, biofeedback, kinesiotaping, pharmacological treatments, physical agent modalities (e.g., ultrasound, TENS, heat application, laser therapy) and invasive techniques such as wet needling and dry needling (DNY) [16,18,29,30]. Specifically, DNY is a minimally invasive treatment technique with a low risk, and is economical, using thin monofilament needles without any drugs (dry needling), inserted at the center of the TrPs.

Therefore, the needle should be inserted perpendicular to the skin, needled backward and forward to the TrPs until no more LTR occurrs [16,31]. Dry needling is typically used to treat muscles, tendons, ligaments, scar tissue, neurovascular bundles and peripheral nerves for the management of a variety of neuromusculoskeletal pain syndromes [29,32].

Several studies support the application of DNY to reduce disability and pain in various pathologies such as hip arthrosis, knee, carpal tunnel syndrome, piriformis syndrome, migraine, temporal mandibular disorder, headache, neck pain, shoulder pain, plantar fasciitis, post-stroke shoulder pain and low back pain [32,33,34,35,36,37,38,39,40,41,42].

The treatment of MTrPs by DNY was first proposed by Lewit, who described a treatment similar to that of Travell and Simons for MTrPs, with the difference being the avoidance of drug injection and the exploitation of only the mechanical effects of the needle to treat myofascial pain, obtaining similar results [29,43,44]. However, the results are highly heterogeneous. In their reviews, Cummings et al. [45] and Tough et al. [46] analyzed the application of DNY in various anatomical segments (trapezius, pectoral, lumbar), showing no differences between DNY and placebo for treating shoulder and lumbar pain. On the other hand, in their review, Kietrys et al. [47] recommended dry needling treatment for cervical pain and shoulder pain, reporting immediate improvement after the end of the therapy session and at four weeks of follow-up. A systematic review by Furlan et al. [48] analyzed the combined use of dry needling and acupuncture for chronic lumbar pain. They observed immediate pain and function improvements in the treated group after treatment and at a close follow-up. Moreover, Abbaszadeh-Amirdehi et al. [49] reported an improvement in clinical outcomes after a single treatment with dry needling of the UT.

Furthermore, Tekin et al. [39] reported an improvement in pain after six sessions of DNY in the treatment of myofascial pain with active MTrP applied to the ISP, trapezius and subscapularis muscles. This heterogeneity could be due to various factors such as the different parameters evaluated, the study sample, the quality of the study, the experience of the therapist, the segment treated, the follow-up considered and the LTR stimulation.

Nonetheless, the effects of DNY have not been fully addressed, and various mechanical, neurophysiological and chemical models have been proposed. To date, the insertion of a needle in the region of the terminal plate can lead to an increase in the discharge of motoneuron, expressed by LTR, reducing the reserves of acetylcholine, increasing microcirculation and the consequent oxygenation. In addition, the reduction in pain shows a double mechanism, both at the periphery through the release of opioids and Cyclooxygenase-2 (COX2), and centrally through the gate control tehory and serotonin release [26].

In light of this evidence, DNY has been utilized in different sport rehabilitation contexts [50,51]. Thompson et al. [50] examined Swedish elite track and field athletes with history of longstanding musculoskeletal pain, reporting the satisfaction of athletes due to the rapid resolution of pain and the immediate return to competition; on the other hand, Fleckenstein et al. [51] reported the decrease in myofascial pain after DNY treatment in various types of athlete, e.g., runners with tendinopathy, military athletes with chest pain, shoulder pain in a non-professional athletes, knee pain in elite ballet dancers, hamstring strain in a pole-vaulters or shoulder pain in three tennis players.

Nonetheless, shoulder injuries in overhead sports can be serious and potentially career-threatening; therefore, it is important to develop strategies to improve the health of athletes (in terms of muscles, tendons, and collagen), achieving an adequate performance through a multidisciplinary rehabilitation treatment [51,52,53].

To date, there is still no consensus on the treatment of myofascial shoulder pain with DNY in the overhead sport population.

Therefore, through the present systematic review, we aimed to evaluate the current evidence on the effect of DNY on myofascial shoulder pain in overhead athletes.

## 2. Materials and Methods

### 2.1. Search Strategy

This systematic review was conducted and reported according to the Preferred Reporting Items for Systematic Reviews (PRISMA) statement [54]. Our searches involved three electronic databases: PubMed, Scopus and Web of Science, from inception to 8 March 2024, following the search strategy, as depicted by Table 1.

The systematic review protocol was submitted to the International Prospective Register of Systematic Reviews (PROSPERO) CRD42024532806. The protocol was not prepared.

### 2.2. Selection of Articles

We adopted the PICO (patient/population, intervention, comparison, outcome) approach: overhead athletes with shoulder pain (P); DNY (I); no treatment, comparison between DNY application, manual therapy and other kind rehabilitation (C); pain, disability, range of motion (ROM), Pain Pressure Threshold (PPT) scores or scales (O). We included randomized controlled trials (RCTs), case–control studies, feasibility studies as the study design, with the full text available and written in English, that met the following inclusion criteria: overhead athletes with shoulder pain, DNY in MTrPs. The exclusion criteria were studies that concerned non-overhead athletes, studies not focused on the treatment of MTrPs with DNY and other reviews.

### 2.3. Data Extraction

Two independent reviewers screened articles by the title and abstract. The full text of the articles selected was reviewed in accordance with the selection criteria. Two reviewers extracted the data from the selected studies on a Microsoft Excel sheet. In case of disagreement, consensus was achieved with a third reviewer. The data extracted from each paper included the following: author and year of publication, subject characteristics (age, sample size, overhead athletes with shoulder pain), description of intervention (DNY) and outcome measures. Data availability on request.

### 2.4. Quality Assessment

We used a modified variant of the STROBE criteria to conduct the methodological evaluation, using ten criteria. Two authors independently assessed the score, and disagreements were evaluated and resolved by consensus. A numerical scoring system (1 if present; 0 if not present) was used to rate the items. Studies were classified as having a high risk of bias if the score was <6 and a low risk of bias if the score was >6.

## 3. Results

### 3.1. Evidence Synthesis

**Figure 1:**
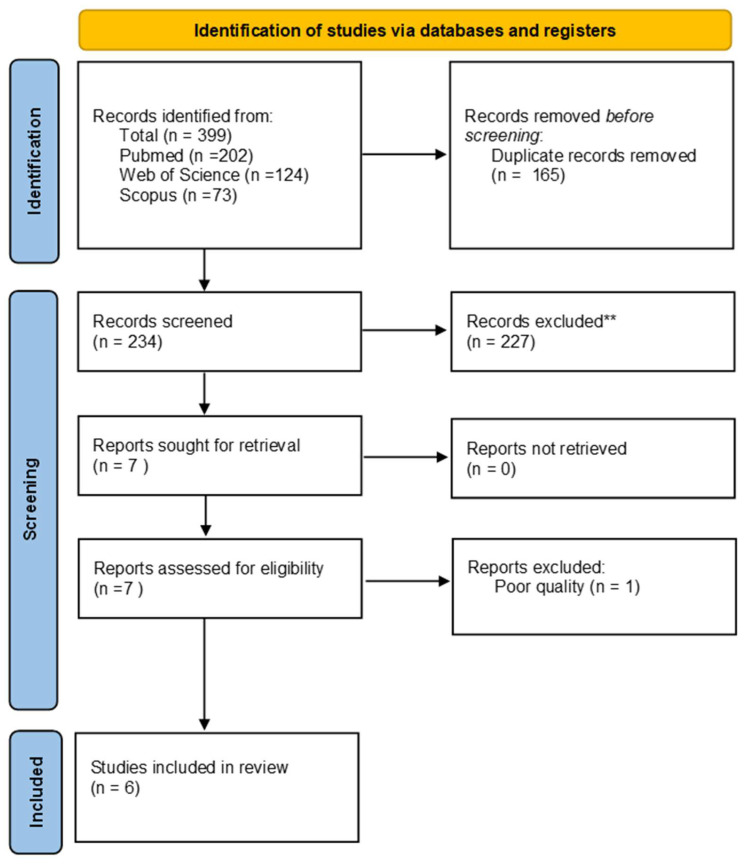
PRISMA flowchart. **: escluded after title and abstract screening.

The literature search on PubMed yielded 202 items, Scopus 73 and Web of Science 124. Out of 399 articles, 165 were excluded because they were duplicates, and 234 articles were screened. Only seven articles met the inclusion criteria. After quality assessment, one article was excluded [55], resulting in a total of six studies included in the systematic review (Figure 1).

### 3.2. Synthesis of the Results

The included studies comprised a total of 145 patients between 18 and 60 years, including 96 men and 49 women. A summary of the included trials is shown in Table 2. Four of the six included trials were RCTs [16,17,23,56]. One study reported two sessions of DNY [57], two studies three sessions of DNY [16,56], and lastly, three studies only one session [17,22,23]. Four studies associated DNY with other treatments (manual therapy, strengthening exercises, diacutaneous fibrolysis (DF), stretching) [22,56,57]. Nevertheless, all studies evaluated pain, and all outcomes were assessed at a short-term follow-up. Four studies reported the size of the needle used [16,17,22,57].

### 3.3. Intervention Protocol

There was a wide range of diversity in the rehabilitation methods in terms of the number of DNY sessions, muscle target and time to outcome. Kheradmandi et al. [56] evaluated the effectiveness of DNY+ manual therapy (MT) in three sessions, each 3 days apart, compared to MT surgery alone, in patients with shoulder pain (≥3 NRC), dyskinesia and MTrPs. The muscles affected by DNY treatment were those with scapular action: subscapularis, pectoralis minor, serratus anterior, UT and lower trapezius. The outcome variables were measured at baseline and immediately after the last session.

Ceballos et al. [17] evaluated the acute effect of a single DNY treatment in elite professional handball (HB) players with MTrPs compared to the control group without any treatment. DNY was performed while US-guided for better targeting, using 0.30 mm × 50 mm needles. In their case study, Javed et al. [57] evaluated the effect of two sessions of DNY, performed after 2 days, combined with heating packs, progressive resistance exercises and aerobic training, starting at the first session and lasting for 12 days from the last session. The outcome variables were measured at the baseline, after each treatment and after 2 weeks, using 0.25 mm × 50 mm needles.

Kamali et al. [16] compared the effect of DNY on ISP in athletes with positive impingement, MTrPs and shoulder pain (≥3/10 Vas) to UT DNY in three sessions separated by 3 days. The outcome variables were measured at baseline and 3 days after the last session, using 0.2 mm × 50 mm needles.

In their case study, Osborne et al. [22] compared the effect of DNY in four volleyball players. A single session was required in three cases, and two sessions on consecutive days in one case. The muscles treated were ISP and teres minor in two cases, and ISP, teres minor and anterior deltoid in the other two cases. The authors used 5 to 12 needles inserted perpendicularly into the affected muscle, and each needle was twisted until tenderness localization, and left in-site for 10 min. In this scenario, DNY was also associated with other forms of treatment such as post-exercise ice therapy, stretching and exercises. Outcome variables were measured at baseline and immediately after treatment, while functional pain was measured at baseline, 2, 3 and 7 days after treatment, using 0.25 mm × 40 mm needles.

Jiménez-del-Barrio et al. [58] evaluated the acute effect of a single DNY treatment in elite professional handball (HB) players compared to diacutaneous fibrolysis, in teres mayor muscle. The outcome variables were measured at baseline, immediately after treatment and after 1 week, using 0.25 mm × 50 mm needles. None of the studies reported any side effects, although pain during the procedure and post-injection soreness, which can last for days, were noted.

### 3.4. Outcome Measures

Pain was evaluated through the validated scales VAS [16,57,58], NRS [17,56] and McGill Pain questionnaire [22]. Nonetheless, the PPT was evaluated using an algometer [16,56]. The disability of the upper limb was assessed by the DASH scale [16,56]. The ROM through a digital inclinometer [17,23,57] and goniometer [22,57]. The isometric strength was evaluated by a hand-held dynamometer [17]. SD (Scapular Dyskinesis) was evaluated by the lateral scapular slide test [56]. Lastly, muscle stiffness was evaluated via myotonometry [58].

#### 3.4.1. Pain

Kheradmandi et al. [56] tested DNY+MT vs. only MT; at the end of the treatment, the authors found a significant (<0.001) improvement in NRS for both groups. Kamali et al. [16] assessed two DNY treatments, and at T1, both groups showed significant (<0.001) improvement in VAS: 4.66 ± 2.01 (UT) vs. 4.84 ± 1.74 (ISP). Ceballos et al. [17] evaluated the efficacy of DNY in teres major compared to the control group without any treatment: a significant (<0.001) improvement in NRS only in the DNY group 3.30 (2.02, 4.59) was found at the end of the treatment. Osborne et al. [22], reported a change in VAS; nonetheless, none of the four athletes’ pain scores increased to near pre-treatment levels through continued training and competition. Javed et al. [57], used a rehabilitation program, including DNY + heating pads + muscle strengthening exercises, reporting an improvement in VAS. Jiménez-del-Barrio et al. [58] evaluated the efficacy of DNY in teres major compared to DF: the authors found an improvement in VAS for both groups immediately and after one week, without statistically significant differences between the groups.

#### 3.4.2. PPT and Disability

Upper limb disability was evaluated using the DASH score. Kamali et al. [16] reported that at the end of intervention, there was a significant (*p* < 0.001) improvement for both groups, with a 20.76 ± 21.92 (UT DNY group) vs. 25.00 ± 19.04 (ISP DNY group). Kheradmandi et al. [56] reported a significant (*p* < 0.02) improvement in the DASH questionnaire at the end of the treatment for both groups: 28.51 ± 5.5 (DNY group) vs. 12 ± 31.5 (control group).

Moreover, the same studies evaluated PPT. Kamali et al. [16] reported a significant improvement (*p* = 0.020) only for the DNY ISP group 0.36 ± 0.61. Kheradmandi et al. [56] reported a significant increase (*p* = 0.007) for the control group (10.85 ± 6.76), whereas the PPT remained almost unchanged (0.20 ± 15.92) in the DN group, explaining that the DNY treatment, especially in UT, leads to greater post-needling soreness even up to 5 days after DNY, which may also lead to less adherence in some cases.

#### 3.4.3. ROM and Isometric Strength

Javed et al. [57] reported an improvement in the measurements taken with an inclinometer and goniometer, from the first treatment (T1) to the maximum improvement after 2 weeks (T2) (T0: flexion 50°, abduction 40°; T1: flexion 85°, abduction 90°; T2: flexion 135°, abduction 150°; after 2 weeks: flexion 160°, abduction 175°). Moreover, Ceballos et al. [16] reported a significant improvement for the DNY group in internal rotation (IR) and extensibility (Ex) (degree of improvement, IR: 23.6°, with *p* < 0.001 between-group difference; Ex: 13.63°, with *p* = 0.025 between-group difference). No differences were found between or within the groups for the external ER and maximum isometric strength. The control group did not show any significant improvement. Osborne et al. [22] reported improvement in all athletes after a single treatment, with a regain of the full ROM in almost all patients. It is important to remark that the authors measured the data before and after the training sessions or matches. Curiously, during the procedure, patients complained of transient worsening of the symptomatology; in case of not complete resolution of the pain, the patients underwent a second treatment. Lastly, Jiménez-del-Barrio et al. [58] showed an improvement in the outcome measurements taken with an inclinometer in both groups with no statistically significant between-group difference (postintervention change score IR DNY: T1 = 16.40 ± 9.25, T2 = 13.73 ± 7.13; ER DNY T1 = 7.26 ± 17.69, T2 = 6.40 ± 17.05; horizontal adduction (HA) DNY: T1 = 14.53 ± 11.12, T2 = 19.73 ± 9.04. IR DF: T1 = 15.21 ± 7.24, T2 = 14.35 ± 6.46; ER DF T1 = 7.92 ± 10.49, T2 = 8.28 ± 12.97; horizontal adduction (HA) DNY: T1 = 13.57 ± 6.52, T2 = 21.00 ± 10.16).

#### 3.4.4. Shoulder Function

As reported by Kheradmandi et al. [56], scapular dyskinesia improved in 70% of the DNY+MT group compared to only 30% of the MT group. Ceballos et al. [17] reported the empty can test, assessed using manual resistance, were subjectively improved.

#### 3.4.5. Stiffness and Tone

Jiménez-del-Barrio et al. [58] evaluated mechanical muscle properties by MyotonPRO. They found no statistically significant differences in teres major muscle stiffness and tone after a single session of DN or DF at T1 and T2.

#### 3.4.6. Risk of Bias

Four of the six studies considered (66%) were of excellent quality [16,17,23,56], one were of very good quality (33%) [57] and the one remaining (33%) was of good quality [22], as shown in Table 3.

#### 3.4.7. Study Limitations

The need for long-term follow-up to evaluate the medium- or long-term effects of DNY treatment was reported by Kamali et al. [16], Ceballos et al. [17] and Jiménez-del-Barrio et al. [58]; in fact, Ceballos et al. [17] described outcomes immediately after treatment. Additionally, Jiménez-del-Barrio et al. [58] described outcomes immediately and after one week of single treatment. Moreover, other limitations regarding the population studied were pointed out. Kheradmandi et al. [56] report the need to consider the type of sport for an accurate analysis of the results. Ceballos et al. [17] and Jiménez-del-Barrio et al. [58] only included male handball athletes. In Kamali et al. [16], the athletes were not under the same resting or active conditions. In addition, the authors reported that to better evaluate the effect of DNY, it would be useful to include a control group or a placebo, and that it is necessary to stratify patients according to the degree of pain and tissue sensitivity [16]. Ceballos et al. [17] and Kheradmandi et al. [56] concluded that multidisciplinary treatment is needed, including not only physical therapy, but also appraising the core balance, as this could lead to shoulder dysfunction. Kamali et al. [16] also suggested that other objective measures, such as strength and ROM, should be used to assess DNY.

Several studies noted the need for long-term follow-up to evaluate the medium- or long-term effects of DNY treatment [14,15]; other limitations included the need to consider the type of sport for an accurate analysis of the results [47], the inclusion of only male handball athletes [15], and the lack of control or placebo groups [14,15,47]. The authors also recommended that multidisciplinary treatment is needed, including not only manual therapy but also core evaluation, as this plays an important role in shoulder dysfunction [14,15,47].

## 4. Discussion

Overhead athletes often experience changes in shoulder range of motion (ROM), particularly with internal rotation (IR) deficits, during the sports season [59]. These changes, closely associated with alterations in eccentric strength, isometric strength and the external/internal rotation strength ratio, represent significant risk factors for the development of shoulder pain and have a substantial impact on performance [60,61,62]. The present review investigated the effectiveness of DNY treatment in overhead athletes with myofascial shoulder pain.

The major findings of this systematic review reported a significant reduction in pain as assessed by the VAS or NRS clinical scales. In particular, the analgesic effect after a single session resulted in a decrease in substance P (SP) in the peripheral site, responsible of the nociceptive stimulation following repetitive microtrauma [26]. Although, there was conflicting evidence in the literature on the effectiveness of DNY in other areas of the body [52,63,64,65,66,67,68], there is a broad consensus on the benefits of DNY in treating shoulder and cervical pain [39,49,69]. The DNY technique could play a key role; inasmuch, Hong et al. highlighted how important it is to stimulate the LTR during treatment with DNY to achieve a valuable effect, as this is an objective confirmation that the needle is in the MTrPs, with a bigger increase in the level of B-endorphin release and SP decreased [26,40,44]. However, there is still no consensus on the minimum number of and interval between treatments. Some studies have used weekly sessions of DNY and it has been suggested that a week is needed between treatments to allow the muscle to recover [70]. Nevertheless, Osborn et al. proposed the treatment in two consecutive days. Furthermore, Kheradmandi et al. and Kamali et al. recommended, respectively, a 2 or 3 times a week treatment, suggesting that overhead athlete DNY therapy can be relatively short, providing brief-term symptom relief.

For athletes, the period out of competition represents a critical factor, and it is mandatory for the medical staff to find treatments that might provide immediate pain decrease, preventing periods of inactivity with few adverse events (AEs). In this context, DNY might produce post-puncture pain, syncopal reactions and bleeding at the injection site, however with relatively few and minor occurrences, and not reported within our review population [29].

However, it is important to consider the PPT assessment, which could be due to post-injection soreness caused by the procedure. In details, as reported by Vegas et al. [71], the intramuscular oedema caused by the practice determines the tenderness, and engages the region affected by the injection and not in the surrounding area. Nevertheless, Osborne et al. [22] tested the effectiveness of the treatment during the sport season, even close to matches or training sessions, showing a positive effect on pain and performance. On the contrary, Kamali et al., Kheradmandi et al., Jiménez-del-Barrio et al. and Ceballos et al. did not specify whether the athletes were training during the season or off-season period [16,17,56,58]. Nonetheless, Thompson et al. [50] showed that athletes appreciated the rapid analgesic effect of the DNY treatment, with a positive influence on reducing the intake of drugs. This is particularly relevant in elite athletes, who have a predisposition to use pharmacological analgesics prior to training and competition up to four times more than the general population of the same age [72,73]. Paracetamol and non-steroidal anti-inflammatory drugs (NSAIDs) are among the most common groups of pharmacological substances used, ranging from 11% to 92%. This could have an impact on performance as, in addition to the known side effects of NSAIDs, they have been reported to interfere with muscle hypertrophy and strength gains in response to chronic resistance training.

The possibility of pre-competition use of DNY is a great advantage, as it is a doping-free procedure with no contraindications to competition. However, physicians should consider post-injection soreness and pain during the procedure, which may lead to poor compliance in athletes. Nevertheless, it is not easy to directly assess the effect of DNY, because it is often part of a broader rehabilitation program. Only Ceballos et al. [17] applied DNY alone, in a single session, and assessed the effect on pain immediately after treatment, obtaining encouraging results. However, a complete analysis of the athletes’ deficits is recommended to plan a tailored rehabilitation program that could integrate DNY to treat MTrPs. Multidisciplinary treatment is needed, including not only manual therapy, but also core evaluation, as this plays an important role in shoulder dysfunction [14,15,47].

This review is not free from study limitations. Firstly, we only included overhead athletes, which could affect the generalizability of the results. Moreover, it is important to consider the type of sport for an accurate analysis of the results [47], or the inclusion of only male handball athletes [15], or the lack of control or placebo groups [14,15,47]. Furthermore, the results could be influenced by the heterogeneities of the protocols, as well as the different control groups. Additionally, only short-term benefits were assessed, and several studies noted the need for a long-term follow-up to evaluate the medium- or long-term effects of DNY treatment [14,15]. Also, the time of the season in which the athlete is participating is not specified in most studies; this is an important variable that should be investigated to better understand if DNY can fit into treatment protocols during periods of high-intensity seasons.

## 5. Conclusions

Taken together, our study showed that DNY might play a role as an effective, rapid and safe treatment of shoulder pain, considering the negative impact that MTrPs could have on overhead player. In particular, DNY could be a useful approach for practitioners during the sports session for shoulder pain management. Nonetheless, further studies are needed to assess the effects on other parameters such as strength, range of motion, muscle elasticity and the long-term effects of DNY.

## Figures and Tables

**Table 1:** Search strategy.

*PubMed* ((dry needling) OR (dny)) AND ((sport) OR (overhead athletes)) AND ((pain) OR (trigger point)) *Scopus* TITLE-ABS-KEY (“dry needling” OR “dny”) AND (“sport” OR “overhead athletes”) AND (“pain”OR “ trigger point”) *Web of Science* (“dry needling” OR “dny”) AND (“sport” OR “overhead athletes”) AND (“pain”OR “ trigger point”)

**Table 2:** Main characteristics of the trials included in the present review.

Author	Design	Patients	Inclusion Criteria	TG	CG	Outcome	Main Finding
Kheradmandi et al., [56], 2021	RCT	N = 40 15 M/25 F Age: 18–45 y.o. DNY group = 32.2 ± 8.35 y.o.; Control group = 31.80 ± 6.85 y.o.	- Trigger points in scapulohumeral muscles - Scapular dyskinesia with test - NRS ≥ 3	Scapular mobilization at 3 sets of 10 repetitions with a 30 s rest between each set + DNY Three sessions for each patient with the interval of every three days	Only MT	NRS; DASH; PPT; SD	Reduction in pain (*p* < 0.001) and disability (*p* = 0.02) with significant improvement in scapular dyskinesia in treatment group (*p* = 0.02). PPT significantly increased in the control group (*p* = 0.004). No adverse effects reported by the participants during this study.
Ceballos et al. [17], 2021	RCT	N = 30 M; Age: 22.39 ± 3.73 y.o. DNY group = 22.47 ± 3.04 y.o.; Control group = 22.31 ± 4.37 y.o.	- Unilateral shoulder pain during throwing actions - 18–30 y.o,—minimum of 2 years’ experience practicing HB - Practice routine of a minimum of 2 h/d and 3 d/w, a GIRD value ≥ 15° - Presence of an active MTrP in the teres major muscle	DNY teres major	No intervention	NRS; ROM (inclinometer); Isometric strength (hand-held dynamometer); Extensibility (inclinometer)	Improvement in pain. External rotation and internal rotation in the DNY group.
Javed et al., [57], 2020	Case study	N = 1 M Age: 22 y.o.	- Male athlete with severe shoulder pain during competition	DNY supraspinatus + heating pads to enhance the blood supply of the area and for analgesic effect + Progressive resistance training exercise	None	ROM (goniometer and inclinometer); VAS	Improvement in ROM and pain (VAS).
Khamali et al. [16], 2019	RCT	N = 40; 20 M/20 F; Age: 18–60; 36 ± 16 y.o.	- Shoulder impingement - Active MTrPs in both UT and ISP muscles - 3/10 VAS	DNY ISP	DNY UT	VAS; PPT; Dash	Pain and disability decreased significantly in both groups. Ppt increase only in infraspinatus.
Osborne et al. [22], 2010	Case study	N = 4 F Age: 25 ± 2 y.o.	- Female volleyball players with recent onset anterior/anterolateral shoulder pain	1 DNY session (subject 2 receive 2 sessions of DNY) + post-training icing, exercises and stretching.	None	ROM (goniometer); McGill Pain Questionnaire; Verbal pain score	Improvement in ROM and functional pain scores.
Jiménez-del-Barrio et al. [58], 2023	RCT	N = 30; 30 M; Age: 25.83 ± 5.39 y.o.	- Elite male handball athletes with reproducible shoulder pain during throwing actions - Between 18 and 30 y.o. - A GIRD value > 15° - A minimum of 2 years as an elite athlete, and a practice routine of a minimum of 2 h/day and 3 days/week - Presence of an active myofascial trigger point (MTrP) in the teres major muscle	1 DNY session	1 DF session	ROM (inclinometer); Muscle stiffness	No between-group difference.

Abbreviations: DNY = dry needling, MT= manual therapy, DASH questionnaire = disability of the arm, shoulder and hand, VAS = visual analogue scale, ROM = range of motion, PPT = pain pressure threshold, SD = diskynesia, NRS = numerical rating scale, MTrP = myofascial trigger points, RCT = randomized controlled trial, y.o. = years old, M = male, F = female, N = number of patient, UT = upper trapezius, ISP = infraspinatus, DF = diacutaneous fibrolysis, CG = control group, TC = treatment group.

**Table 3:** Risk of bias.

Article	Criteria for the Quality Scoring										Score
	1	2	3	4	5	6	7	8	9	10	
Kheradmandi et al. [56], 2021	1	1	1	1	1	1	1	1	1	1	10
Ceballos et al. [17], 2021	1	1	1	1	1	1	1	1	1	1	10
Javed et al. [57], 2020	1	1	0	1	1	0	1	0	1	1	7
Khamali et al. [16], 2019	1	1	1	1	1	1	1	1	1	0	9
Osborne et al. [22], 2010	1	1	0	1	1	0	1	0	1	0	6
Jiménez-del-Barrio et al. [58], 2023	1	1	1	1	1	1	1	1	1	1	10

Abstract informative and balanced (1); presence of detailed objectives, incorporating the hypotheses of the study (2); availability of eligibility criteria (3); for the variables of interest, availability of the sources of data and characteristic of the measurement methods, and description of the method correspondence (when there are two or more groups) (4); quantitative variable (5); summarized characteristics of the study population (6); key results focus on the study aim (7); declare limitations (8); careful interpretation of the results based on the objectives, similar literature and other relevant evidence (9); funding statement (10).
